# Hemangiopericytoma of the Breast: A Case Report and a Review of the Literature

**DOI:** 10.1155/2015/210643

**Published:** 2015-01-22

**Authors:** Georgios Koukourakis, Evagelos Filopoulos, Kasiani Kapatou, Georgios Zacharias

**Affiliations:** ^1^Radiation Oncology Department, Saint Savvas Anticancer Institute of Athens, Alexandra's Avenue 171, 11522 Athens, Greece; ^2^Breast Surgery Department, Saint Savvas Anticancer Institute of Athens, Alexandra's Avenue 171, 11522 Athens, Greece; ^3^Clinical Pathology Department, Saint Savvas Anticancer Institute of Athens, Alexandra's Avenue 171, 11522 Athens, Greece; ^4^Laboratory of Hematology, Panarcadian General Hospital of Peloponnese, Red Cross Street, 22100 Tripoli, Greece

## Abstract

*Introduction*. Sarcomas of the breast are rare and hemangiopericytoma (HPC) of the breast is even rarer. *Case Report*. We report a case of a 43-year-old woman who presented with a 4 cm mass in her right breast. Her family history was positive for breast cancer. A fine needle aspiration indicated a malignant vascular tumor. An excision biopsy and frozen section analysis confirmed the presence of an encapsulated mesenchymal tumor. Its morphology and immunohistochemical marker profile were characteristic for a malignant hemangiopericytoma. Thus, she underwent a tumor excision without an axilla sampling. Approximately one year after the surgery the patient is well without local recurrence or metastasis disease to be observed. We also reviewed the literature and discuss the treatment options, characteristics, and immunophenotype of HPC. *Conclusions*. The accurate diagnosis of HPC depends on the appropriate histological and immunohistochemical examination. Surgical resection is the treatment of choice and due to scarcity of cases and unpredictable biological behavior of these tumors long term follow-up may be warranted.

## 1. Introduction

The incidence of primary nonepithelial malignancies localized in the breast is very low and comprise less than 5% of all breast neoplasm [1]. Hemangiopericytoma is a rare vascular tumor originating from the capillary pericytes, which are contractile spindle cells surrounding the capillaries and postcapillary venules [2]. It was first described by Stout and Murray in 1942 as Zimmermann's pericytes which proliferate in the vessel wall [3]. They are typically located in the retroperitoneal space, limbs, body, and head neck region but are extremely infrequent in the breast [4]. A case of 43-year-old woman who presented with the unusual diagnosis of hemangiopericytoma of the breast is discussed in this trial. Moreover, a comprehensive literature review focusing on the clinical and pathologic aspects as well as treatment options of hemangiopericytoma of the breast is provided.

## 2. Case Report

On January 10, 2014, a 43-year-old woman was admitted to our hospital with a persistent enlarged mass in her right breast. The mass was painless and without nipple discharge or hemorrhage. Her medical history was unremarkable, whereas her family history discovered that her mother was diagnosed with left breast cancer at the age of 60. On physical examination, the patient had a palpable, soft tumor, approximately 2 cm in diameter mass, located in the upper lateral quadrant of his right breast. It was a well-defined, mobile mass, not adherent to either the skin or chest wall. No axillary lymph nodes were found and all laboratory studies were within normal limits.

**Figure 1:**
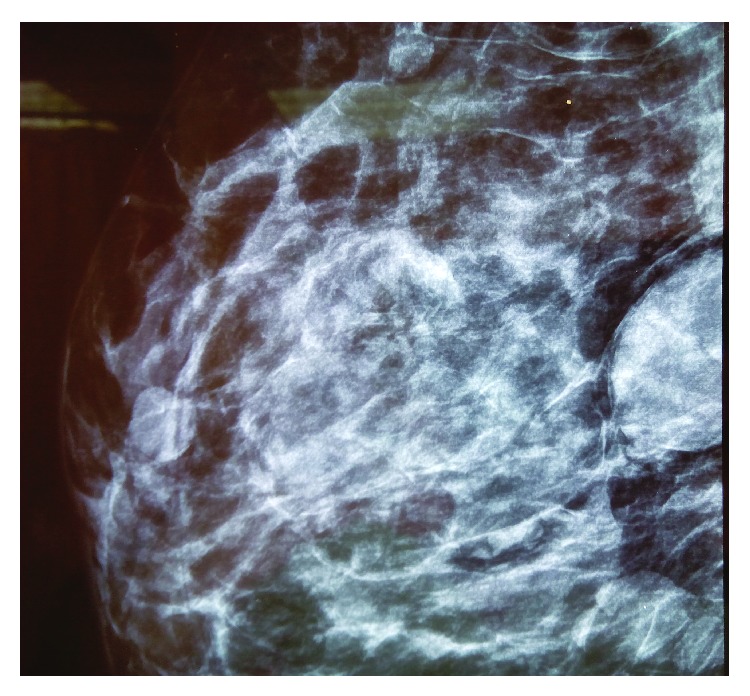
A 28 mm × 34 mm well-circumscribed mass at upper lateral quadrant of the right breast. There was no calcification.

Current mammography revealed a well-defined homogeneous lesion corresponding to the palpable mass of 28 mm × 34 mm in size located in upper lateral quadrant (Figure 1). Targeted breast ultrasonography demonstrated a large heterogeneous and hypoechoic lesion, in the upper lateral quadrant with its largest dimension approximately 3 cm. Fine-needle aspiration was performed and the results were suggestive for mesenchymal tumor. The patient eventually underwent an excision biopsy and frozen section analysis confirmed the presence of an encapsulated mesenchymal tumor of unknown biological potential with positive surgical margins. Subsequently, the patient had a larger tumor excision without sentinel lymph node biopsy.

**Figure 2:**
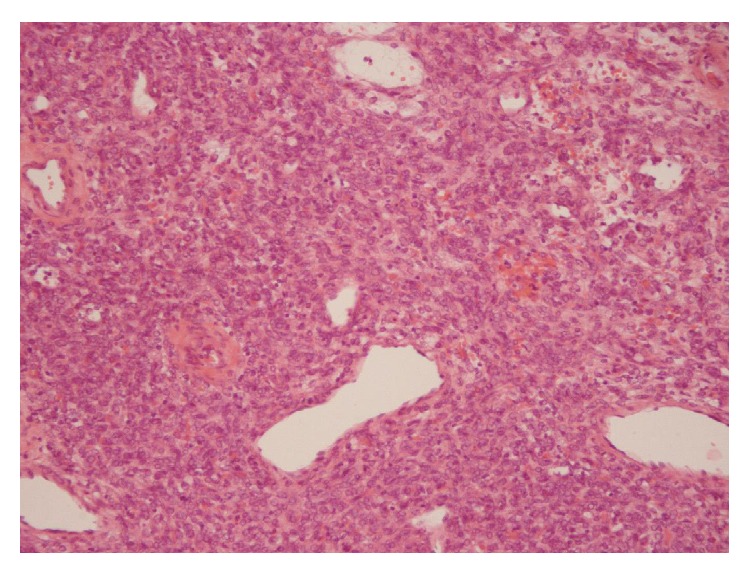
Many round to fusiform tumor cells surrounded the capillaries (Hematoxylin/eosin, ×100).

**Figure 3:**
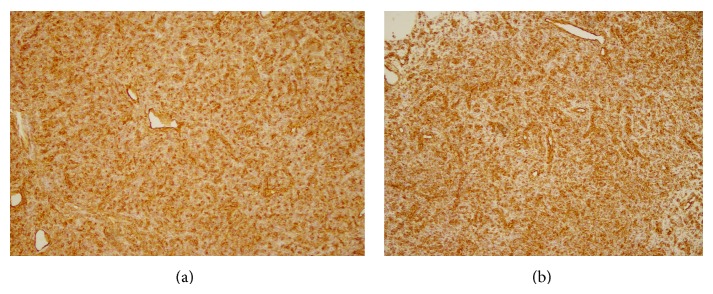
Immunohistochemistry demonstrating the strong expression of the (a) vascular marker CD34 and (b) vimentin by all tumor cells, ×25.

Gross pathology of the resected specimen demonstrated a 4 × 3 × 2 cm measuring, coloured tumor without skin infiltration and containing hemorrhagic fluid. The histological examination of the tumor showed a typical vascular pattern with vessels forming a continuous ramifying network filled with erythrocytes. The tumor cells appeared round to fusiform with a dense reticulin meshwork surrounding the individual tumor cells (Figure 2). The calibre of the vascular spaces varied considerably. Areas of hemorrhage and cystic degeneration were found. The mitotic activity was not significantly increased without necrosis. Affiliated immunohistochemistry was performed. The tumor cells expressed CD34, bcl-2, CD99, and vimentin and were negative for LCA (leukocyte common antigen), desmin, SMA (smooth muscle antigen), CK7, CK34, BE12, CK19, and CK20, (Figure 3). This immunohistochemical expression profile and the morphological aspect were consistent with the diagnosis of a malignant hemangiopericytoma with low grade of differentiation. Moreover, due to the R0-resection, adjuvant radiotherapy was not mandatory and simply meticulous follow-up was advised.

Approximately one year after diagnosis the patient remains well, without tumor recurrence.

## 3. Discussion

HPC of the breast is extremely rare, but its malignant potential is varied. Consequently, the prognosis of this disease is miscellaneous as well [10]. Currently, there are no guidelines available on how to treat a hemangiopericytoma. Based on the literature review we herein discuss some problems concerning the diagnosis and the therapy of this rare disease. The most important trials giving information about HPC of the breast are presented in Table 1.

Generally, there are a large variety in clinical findings regarding the case of breast hemangiopericytoma. The presentation can be marked by a gradually enlarging, firm, well-defined, and painless mass within the breast which is not attached to the skin. Besides the fact that a discoloration can occur, other skin and nipple changes are rarely seen. Potential routes of spread are local extension or hematogenous dissemination whereas the lymphogenous dissemination is rarely seen. The lung is the most frequent metastatic site presented in breast HPC [7].

The biological behavior of HPC is varied and difficult to predict. Morphologically, HPC is generally characterized by spindle cell proliferation showing a patternless architecture and staghorn vasculature. The final diagnosis is based on specific immunohistochemistry characteristics which also help to distinguish it from the solitary fibrous tumors (SFT). CD34 immunoreactivity has been reportedly revealed to be strongly and diffusely expressed in many cases of HPC/SFTs but it is not specific for SFT or HPC alone. Some studies suggest that additional immunoreactivities of bcl2 and CD99 are also diffusely positive in most SFTs. This feature can sometimes differentiate SFTs from HPC, because this spectrum of tumors shares similar histological pattern and CD34 reactivity. Vimentin, keratin, SMA, epithelial membrane antigen (EMA), desmin, CD117, and S-100 protein are sometimes useful for differential diagnosis of HPC/SFT from tumors with muscle, epithelial, or neural origin. We employed a range of antibodies to characterize the immunophenotype of the tumor cells and found that the tumor cells were positive for CD34, CD99, bcl-2, and vimentin. These markers in hemangiopericytoma were also identified by different authors. Kanazawa et al. [7], Sobel et al. [11], and Jimenez-Ayala et al. [12] had also described a positive expression of vimentin in their study.

According to the literature, complete tumor resection with negative surgical margins represents the treatment of choice because surgery is the only potential curative modality [13]. The extent of surgery depends on the size of the tumor and the breast itself. In terms of lymph node dissection, adequate data are lacking as few cases are reported. In general, lymph node metastases are rare. Therefore, complete axillary lymph node dissection does not seem to be necessary [11]. In our case, we performed a wide tumor excision without axillary lymph node dissection whereas other authors suggested that a most radical excision such as a modified radical mastectomy may be necessary [7].

According to the research of Chugh and Baker [1], under some circumstances radiotherapy is recommended, for example, if the tumor is more than 2 cm in diameter, after incomplete resection, or for tumor reduction before surgery. Nevertheless, there is no authoritative evidence to demonstrate that radiotherapy is necessary for HPC of the breast. Moreover, efficiency of chemotherapy in soft tissue sarcomas, including the hemangiopericytoma of the breast, is not well defined [14]. Finally, evolving therapies inhibiting specific angiogenic pathways show promising activities in HPC, whereas the combination therapy with temozolomide and bevacizumab has recently emerged as an encouraging therapeutic regime [15].

In conclusion, due to limited data available on this rare disease, no general therapeutic recommendations exist. The complete tumor excision and the close follow-up based mainly on clinical examination and annual mammography seem to be extremely important. Adjuvant therapy including radiation therapy or chemotherapy has no established role, whereas the combination of evolving therapies inhibiting specific angiogenic pathways such as bevacizumab with temozolomide has recently emerged as an encouraging therapeutic regime.

## Figures and Tables

**Table 1:** The most important trials regarding HPC of the breast.

Author	Year of publication	Tumor location. Sex	Therapy	Immunohistochemistry	Results
Buecker et al. [5]	2008	Left breast. Female	TRM with excision of the pectoral fascia and an axillary lymph node sampling	The tumor cells expressed CD31, CD34, CD117, CD99, and vimentin	6-month postoperative follow-up, no evidence of disease

Tang et al. [6]	2008	Left breast. Female	TRM and an axillary lymph node sampling	Tumor was positive for CD34 and negative for vimentin, desmin, actin, and S-100	NS

Kanazawa et al. [7]	1999	Left breast. Female	Simple mastectomy	Tumor cells were only positive for vimentin	18 months after operation, free of local recurrence or distant metastasis

Wang et al. [8].	2011	Right breast. Male	TRM + 4 axillary lymph nodes	The tumor cells were positive for CD31, CD34, CD99, F-VIII, vimentin, and SMA	9-month postoperative follow-up, no evidence of disease

Dragoumis et al. [9]	2013	Right breast (pectoralis major muscle). Female	Tumor excision	The tumor cells were positive for CD34 and negative for desmin, SMA, and S-100 protein	20-month postoperative follow-up, no evidence of disease

TRM: modified radical mastectomy; NS: not stated; SMA: smooth muscle actin.

## Abbreviations

TRM: Modified radical mastectomy
NS: Not stated
SMA: Smooth muscle actin.
